# Evaluation of the styloid process on digital panoramic radiographs

**DOI:** 10.4103/0971-3026.73537

**Published:** 2010-11

**Authors:** Chandramani B More, Mukesh K Asrani

**Affiliations:** Department of Oral Medicine and Radiology, K. M. Shah Dental College and Hospital, Sumandeep Vidyapeeth University, Piparia, Vadodara, Gujarat, India

**Keywords:** Eagle syndrome, panoramic radiography, stylohyoid ligament, styloid process

## Abstract

**Background:**

The styloid process is an anatomical structure, whose clinical importance is not well understood. Proper clinical and radiographic evaluation can detect an elongated styloid process and calcification of the stylohyoid ligament. It has been reported that 2 – 28% of the general population show radiographic evidence of mineralization of a portion of the stylohyoid chain. The elongated styloid process may be symptomatic in many cases. Panoramic radiography is the best imaging modality to view the styloid process bilaterally.

**Aim:**

To assess the styloid process on digital panoramic radiographs.

**Materials and Methods:**

The study was conducted on 500 digital panoramic radiographs available in the archives of our department as soft copies. These radiographs were taken using a digital panoramic system. The radiographic length of the styloid process was measured on both sides using the measurement toolbars on the accompanying analysis software. For statistical analysis we used the unpaired *t* test, Chi-square test, and one-way ANOVA test, as necessary.

**Results:**

The average length of the left styloid was 25.41 ± 6.32 mm and that of the right styloid was 25.53 ± 6.62 mm. The length of both styloids increased with age and males had longer styloids than females. Elongated styloids were present in 19.4% of the panoramic radiographs. Langlais type I elongated styloids and a partial calcification pattern were more common than others.

**Conclusion:**

Panoramic radiography is useful for detection of an elongated styloid process and / or ossification of the stylohyoid ligament in patients with or without symptoms, and helps avoid a misdiagnosis of tonsillar pain or pain of dental, pharyngeal, or muscular origin.

## Introduction

The styloid process is a cylindrical bone that arises from the temporal bone in front of the stylomastoid foramen.[1] It normally measures about 25 mm in length, although it varies in length from person to person and even from side to side in the same person.[2,3] Studies have estimated that in 2 – 28% of the general population there is radiographic evidence of an elongated styloid process, although symptoms are present in only some individuals.[4] When symptoms are associated with elongation of the styloid process, the condition is termed as an Eagle syndrome.[3]

## Materials and Methods

A total of 500 digital panoramic radiographs, which were available as soft copies in the hard drive of the computer in our Radiology Department, were selected for the study. Only those radiographs showing the styloid processes of both sides were included, while radiographs having positioning and magnification errors were excluded during this selection process. These radiographs were taken with a digital panoramic system (Kodak 8000C, Mumbai, India) under standard exposure factors, as recommended by the manufacturer.

**Figure 1:**
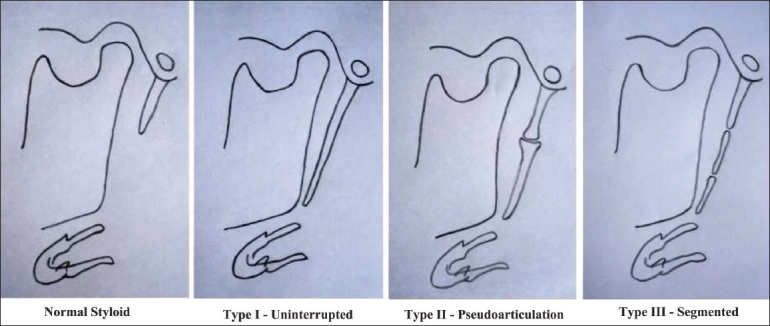
The diagram shows Langlais classification of the type of elongation of the styloid process

The selected radiographs were of patients above 18 years of age. The apparent length of the styloid process was measured with the help of the measurement tools on the accompanying software (Kodak, version 6.7, Mumbai, India). The magnification factor used for the machine was 1.29. The length of the styloid process was measured as the distance from the point where the styloid process left the tympanic plate to the tip of the process, regardless of whether or not the styloid process was segmented. Styloid processes measuring more than 30 mm were considered as elongated.[5] If the stylohyoid or stylomandibular ligaments were ossified, they were measured along with the styloid process, as part of the elongated styloid process. The type of elongation of the styloid processes was also classified as per Langlais [Figure 1].

The collected data was entered in a spreadsheet (Excel 2007, Microsoft, Richmond, USA) and was analyzed using statistical analysis software (SPSS version 17, Chicago, USA). The chi-square test, unpaired *t* test, and one-way ANOVA were used for analysis.

## Results

The average lengths of the left and right styloids were 25.41 ± 6.32 mm and 25.53 ± 6.62 mm, respectively. The average length of both the styloids showed positive correlation with age [Table 1]. Males had longer styloids than females [Table 2].

**Table 1:** Mean length of the styloid process in the different age groups

Age group (in years)	*n* (%)	Average length of the styloid (mm)	
		Left side	Right side
< 20	31 (6.2)	22.75 ± 4.45	23.09 ± 4.16
21 – 30	129 (25.8)	24.50 ± 6.22	24.51 ± 5.76
31 – 40	125 (25)	25.39 ± 5.89	25.49 ± 6.51
41 – 50	97 (19.4)	25.75 ± 7.05	26.15 ± 7.45
51 – 60	66 (13.2)	26.80 ± 5.76	26.57 ± 6.66
61 – 70	38 (7.6)	27.32 ± 7.13	27.26 ± 7.84
> 70	14 (2.8)	25.64 ± 7.18	26.63 ± 7.69
*P* value		0.018 (s)	0.046 (s)

s = Significant

**Table 2:** Mean length of the left and right styloid processes in the two sexes

Gender	*n* (%)	Average length of styloid process (mm)	
		Left side	Right side
Males	242 (48.4)	26.18 ± 6.59	25.90 ± 6.68
Females	258 (51.6)	24.69 ± 5.97	25.17 ± 6.55
*P* value		0.008 (s)	0.21 (ns)

s = Significant; ns = not significant

Out of the 500 panoramic radiographs, 66 showed bilateral elongation of the styloid and 31 showed a unilaterally elongated styloid process (18 on the right side and 13 on the left side). Thus, 97 radiographs (19.4%) showed at least one elongated styloid process.

In the 500 panoramic radiographs studied, a total of 1000 styloid process were evaluated. Out of these, 163 styloids (16.3%) were elongated [84 on the right side (8.4%) and 79 on the left side (7.9%)].

Elongated styloid processes were more prevalent in the age-group of 31 – 50 years and in those > 70 years of age [Table 3].

**Table 3:** Mean length of the left and right styloid processes in the different age groups

Age group	*n* (%)	Average length of styloid process (mm)	
		Left side	Right side
< 20	2 (2.06)	33.20 ± 2.69	33.95 ± 2.49
21 – 30	24 (24.74)	34.11 ± 6.32	33.18 ± 6.43
31 – 40	23 (23.71)	33.97 ± 7.20	35.78 ± 7.50
41 – 50	18 (18.56)	37.05 ± 7.92	38.66 ± 7.44
51 – 60	15 (15.56)	34.47 ± 5.94	35.06 ± 7.51
61 – 70	12 (12.37)	34.88 ± 7.91	35.30 ± 9.14
> 70	3 (3.09)	36.30 ± 6.94	38.67 ± 5.59
*P* value		0.84 (ns)	0.39 (ns)

ns = not significant

**Figure 2:**
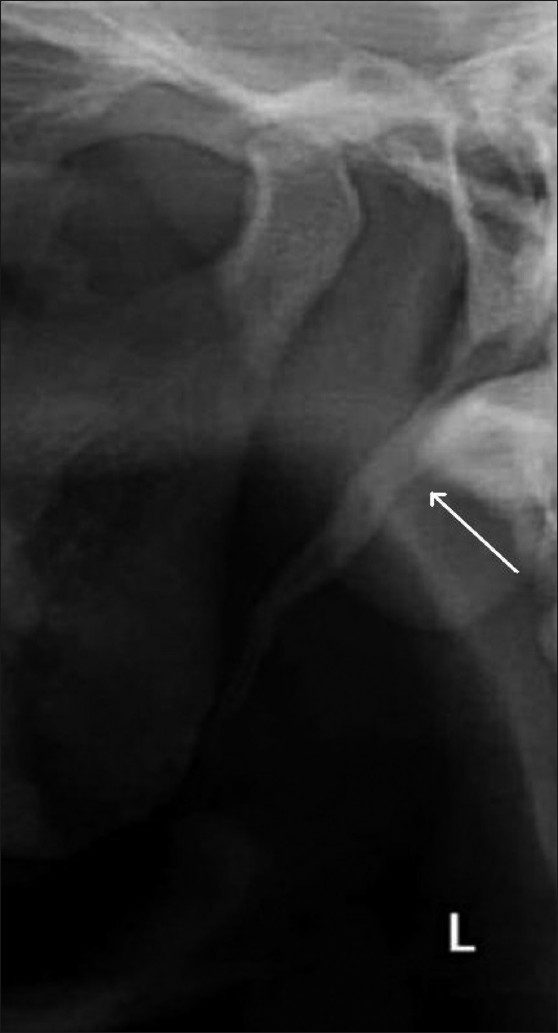
The orthopantomogram shows a Langlais type I styloid process (arrow)

**Figure 3:**
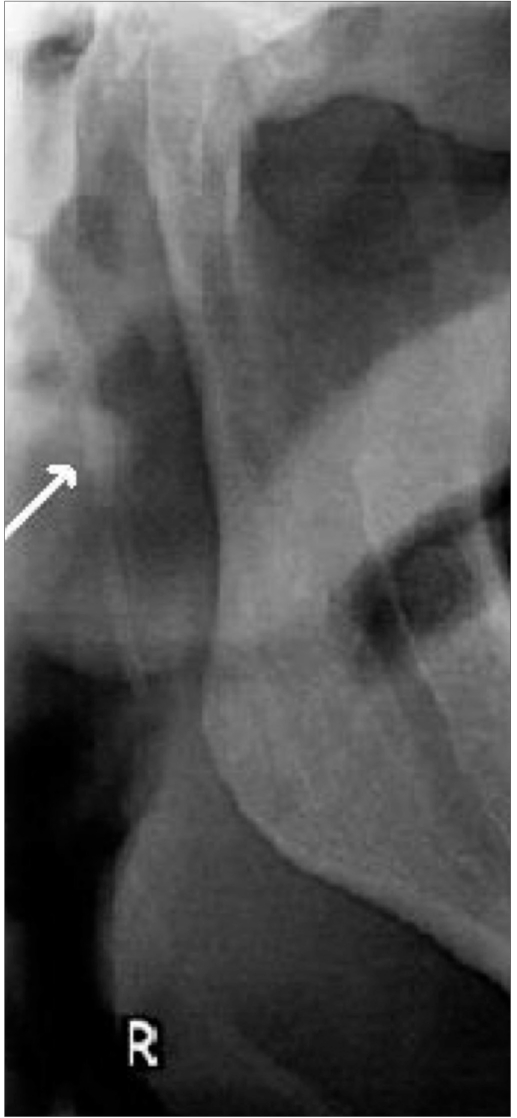
The orthopantomogram shows a partially calcified styloid process (arrow)

**Figure 4:**
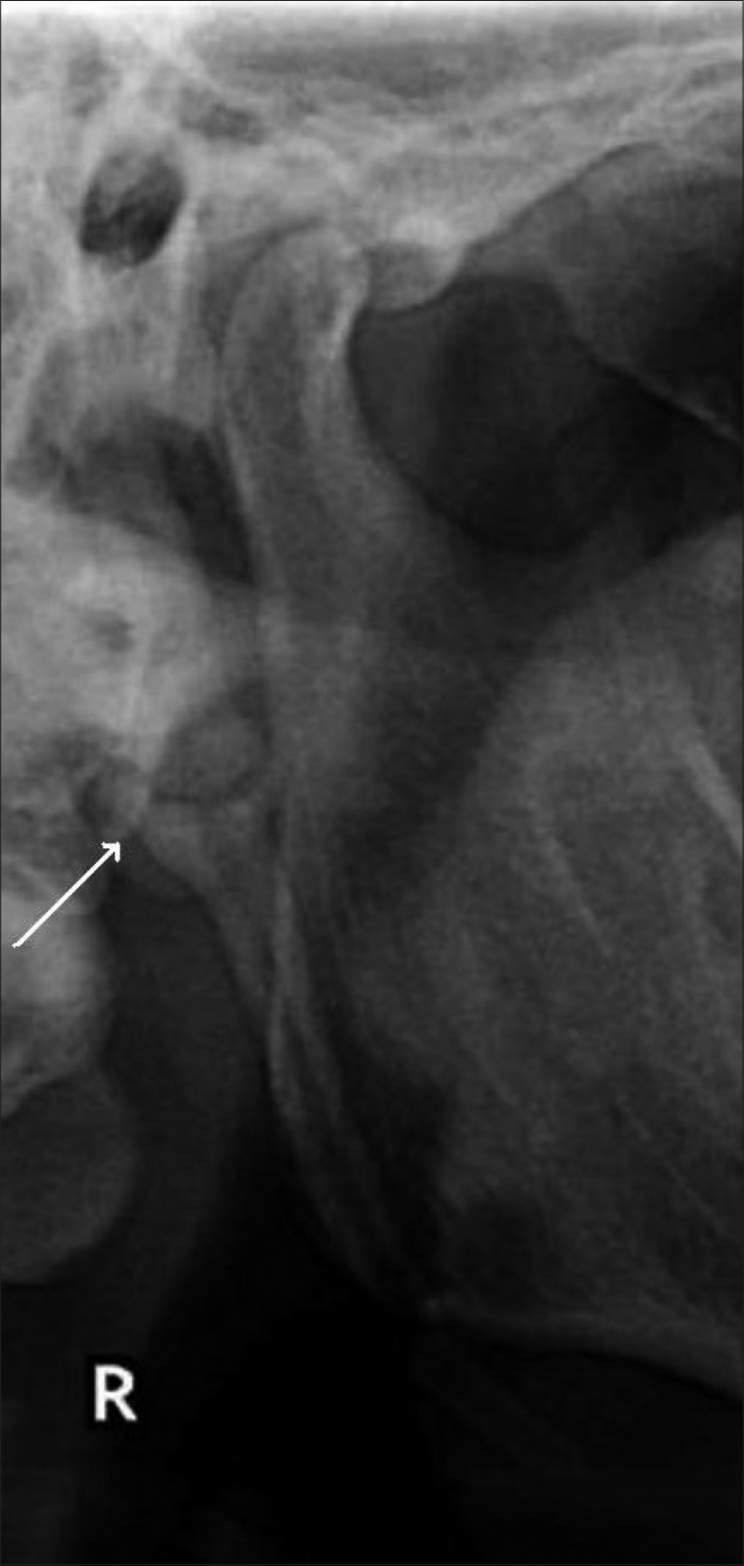
The orthopantomogram shows a Langlais type II styloid process (arrow)

**Figure 5:**
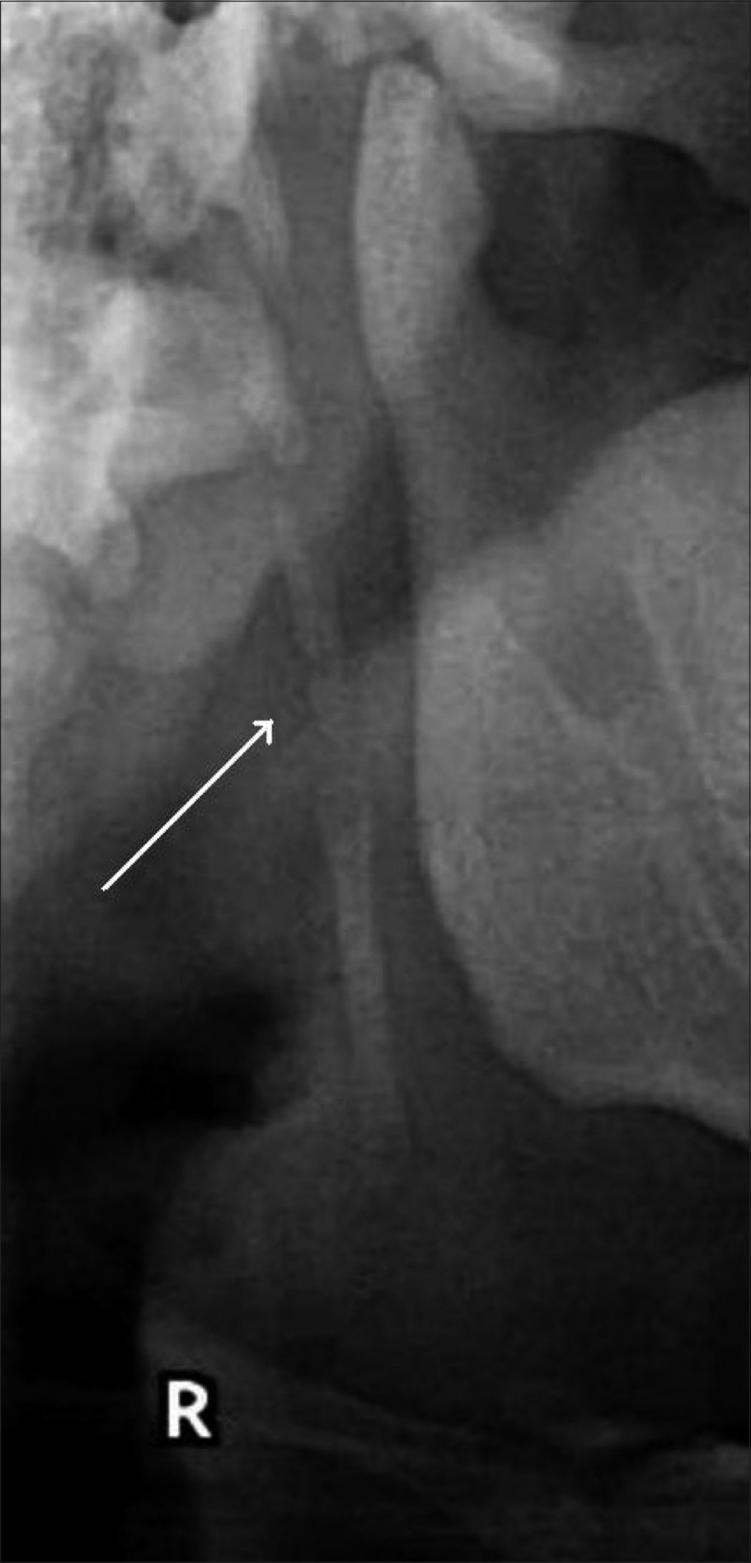
The orthopantomogram shows a Langlais type III styloid process (arrow)

More than 85% of the elongated styloid processes had Langlais type I elongation [Figure 2] and more than 70% had partial calcification of the styloid process [Figure 3]. All the age-groups showed a predominance of Langlais type I elongation, with partial calcification. Langlais type II elongation [Figure 4] was found in 12.65% on the left side and in 7.14% on the right side, and Langlais type III elongation [Figure 5] was seen in 1.26% on the left side and 5.95% on the right side.

## Discussion

Various theories have been proposed to explain the ossification of stylohyoid / stylomandibular ligaments, namely, theory of reactive hyperplasia, reactive metaplasia, anatomic variance,[6,7] and aging and developmental anomaly, due to loss of elasticity in the ligament simulating tendinosis.[8]

Langlais (1986)[9] has classified elongated styloid processes according to the type of elongation [Figure 1].

Among the several imaging modalities used for diagnosis of the Eagle syndrome, panoramic radiography, lateral skull radiograph, Towne’s view radiograph, anterioposterior skull radiograph, and CT scan are some of them. The complete details of the length, angulation, and relation to adjacent structures can be obtained from a CT scan by formulating a 3D-CT.[10,11]

In the present study, the average length of the left and right styloids were 25.41 ± 6.32 mm and 25.53 ± 6.62 mm, respectively. Eagle[12] has reported that the normal styloid process measures 2.5 – 3 cm, whereas, Kaufman *et al*.[5] reported 30 mm as the upper limit for the normal styloid process. Various investigators have reported the incidence of elongated styloid as 1.4, 4, 7, and 18.2%, respectively.[5,12–14] Additionally, in our study, we noted radiographic mineralization of the stylohyoid ligament in 19.4% of the panoramic radiographs.

There is a progression in the length of calcification with advancing age.[6] Our findings were similar to those obtained in different studies by other investigators.[15] We also noted that males had longer styloids as compared to females. However, this finding differed from those of some other researchers, who found an increased incidence in females.[15] Our study also showed unilateral elongation of the styloid in 31.95% and bilateral elongation in 68.05% of the panoramic radiographs. Bozkir *et al*. had noted unilateral elongation in 25% and bilateral elongation in 75% of the panoramic radiographs.[4]

We further observed that more than 85% of the elongated styloid processes on the panoramic radiographs had Langlais type I elongation – 86.07% on the left side and 86.7% on the right side. Langlais type II elongation was found in 12.65% on the left side and in 7.14% on the right side, and Langlais type III elongation was seen in 1.26% on the left side and 5.95% on the right side. Bozkir *et al*.[4] studied panoramic radiographs of 200 edentulous patients, above 50 years of age, and found elongated styloid processes in eight patients; among these eight patients, two had unilateral and six had bilaterally elongated styloid processes. The average length of the elongated processes in their study was 53 mm. They reported that 42% were of uninterrupted type I and 58% were of interrupted type III. This difference in the results of our study may be due to differences in the age structure and sample sizes.

## Conclusion

Panoramic radiography is useful for detection of an elongated styloid process and / or ossification of stylohyoid ligaments in patients with or without symptoms and can thus help avoid misinterpretation of the symptoms as tonsillar pain or pain of dental, pharyngeal, or muscular origin.

Due to the medial angulation of the styloid process and superimposition of other skeletal structures, some errors may occur when measuring the length of the styloid. Further imaging studies are required to correlate the symptoms with an elongated styloid process as well as with the type and pattern of elongation of the styloid process.
